# Seeing with new eyes: The essence of creativity

**DOI:** 10.1002/cns3.20066

**Published:** 2024-03-17

**Authors:** E. Steve Roach

**Affiliations:** ^1^ University of Texas at Austin Dell Medical School Austin Texas USA

**Keywords:** music, history, Hower Award

The real voyage of discovery consists not in seeking new lands, but in seeing with new eyes.

Marcel Proust

The most memorable presentations at the Child Neurology Society's annual meeting are typically the award lectures. The society's awards recognize substantially different spheres of achievement, so the award lectures differ greatly in their approach and focus. The Hower Award honors an individual with a record of service to society and substantive contributions to the field. The Sachs Lecturer delves deeply into a scientific topic of current interest. The Dodge Award recognizes a promising early career researcher, and the recently added Denkla Award highlights contributions within the field of human development. Each award lecture is unique, but together, they illustrate what makes child neurology such a remarkable field.

As extraordinary as these award lectures have been, only a few have been developed into publications. Most have simply vanished, leaving nothing more than the awardee's name in an archival list of prior award winners. These presentations provided an annual snapshot of the developing field, but we did not do a very good job of preserving them. One of the goals of *Annals of the Child Neurology Society* is to publish articles derived from the society's award presentations. Some oral presentations lend themselves to print conversion better than others, of course, so the aim is to capture the essence of each lecture rather than a mirror image of the meeting presentation.

This issue of *Annals of the Child Neurology Society* contains our first award‐related article, a captivating discussion of the neurology of creativity by Phillip Pearl based on his 2023 Hower Award presentation in Vancouver.^1^ Dr. Pearl knows a great deal about creativity, whether applied to scientific discovery or to his long‐standing passion for music. But in the article, he also explores the thought patterns that promote creativity and delves deeply into its neurophysiologic basis. I attended Dr. Pearl's Hower Award lecture last year, but reading his article allowed me to grasp some of the finer points that escaped me during the presentation.

Pearl's splendid article also perfectly illustrates why we need to remember and preserve the society award lectures. These superb presentations remind us of the soaring heights we as a profession can achieve. They allow us to gauge our progress over time. They should be preserved and become part of our legacy.

## Author Contributions

E. Steve Roach: Conceptualization; project administration; writing–original draft; writing–review editing.

## Conflict of Interest Statement

The author is the editor‐in‐chief of the *Annals of the Child Neurology Society*. The opinions expressed in this article are those of the author and do not reflect the official policy of the Child Neurology Society.
